# Supplementary material for: Sex and age differences in the association between routine suicide newspaper reporting and change in admissions of suicidal patients: An investigation at an emergency and critical care center in Tokyo
Source: PLoS One. 2025 Aug 18;20(8):e0330343. doi: 10.1371/journal.pone.0330343 (PMC12360520; doi:10.1371/journal.pone.0330343)
Supplement: Attachment — Submitted filename: Response to Reviewers.docx [file pone.0330343.s006.docx]

**RE: PONE-D-24-46119** Sex and age differences in the association between routine suicide newspaper reporting and change in admissions of suicidal patients: An investigation at an emergency and critical care center in Tokyo

**Our responses to comments by reviewers:**

Reviewer #1:

Comment 1-1

My only question about the context of this study was whether young people in Japan still read newspapers or get their news from other media outlets such as social media and how that could be more of an impact/ be a confounding factor especially because newspapers as you have noted do follow WHO recommendations, but other sources of information might not.

Response 1-1

The subscription rate of newspapers is an important indicator of their influence. In Japan, subscription rates have been gradually declining, particularly among younger generations, making it necessary to include these figures in this paper. According to the report titled *2015 National Media Contact and Evaluation Survey Report* conducted by *The Japan Newspaper Publishers & Editors Association* as part of the *National Survey on Media Usage and Evaluation*, the overall subscription rate in 2015, which was approximately the midpoint of the study period (April 2012 to January 2019), was 77.7%. Meanwhile, the subscription rate among those in their 30s, close to the median age group of admitted patients in this study, was 40.5%. Subscription rates across all generations have been declining year by year. For instance, in 2021, the overall subscription rate and the subscription rate among those in their 30s were 60.9% and 32.4%, respectively. Some of these figures are incorporated into the main text of the paper.

In 2015, approximately the midpoint of the study period, the newspaper subscription rate in Japan was reported to be 77.7% across all age groups and 40.5% among those in their 30s. (31) (Lines 113-115)

As noted by the reviewer, SA (suicide attempt) occurrence is thought to be influenced by the level of adherence to WHO recommendations. However, in this study, the adherence status of media other than newspapers has not been clarified, necessitating some reservation in interpreting the results. This has been added to the limitations as a potential complicating factor.

The suicide reports from other media may differ in nature from those of newspapers, particularly in terms of adherence to the WHO media recommendations. (Lines 605-607 in Limitations)

Reviewer #2:

Comment 2-1

It would be helpful to include a more detailed explanation of how the specific suicide methods reported in the newspapers were categorized and analyzed. This would provide clearer insight into how these methods were considered in the context of their impact on different populations.

Response 2-1

SA methods selected for analysis included the most frequent seven suicide methods among the 771 reported suicide decedents. The sentence below is inserted in Revised manuscript.

Assessed suicide methods included Hanging, Railroad jumping, Jumping from a height (Height jumping), Gas poisoning, Firearm discharge, Burning, and Cutting or stabbing. For analysis, the items used were the most frequently observed methods among reported suicide decedents, Other methods, and No method reported. (Lines 138-142)

Comment 2-2

While the statistical analyses are robust, further elaboration on the choice of nonparametric tests and why they were preferred over parametric tests for this dataset would enhance the transparency of the methodological approach.

Response 2-2

MANOVA is an appropriate analytical method for examining the relationships between interrelated dependent variables and independent variables. Furthermore, in this study, since the matrix of dependent variables is singular (The sum of ΔFemale and ΔMale equals the sum of ΔYounger and ΔOlder.), the nonparametric MANOVA was required for the analysis. The following sentence has been added to the manuscript:

The nonparametric MANOVA model was crucial for evaluating the associations while accounting for the interrelationships among the dependent variables. More importantly, it resolved the issue of matrix singularity among these variables in the analysis. (Lines 203-206)

Comment 2-3

The authors do an excellent job of linking their findings to psychological and sociocultural factors, but they might want to expand on the implications for future suicide prevention programs, especially considering the differential effects observed across sex and age groups.

Response 2-3

In response to this comment, we have organized and presented a discussion on the possible differential impacts for each sex and age group population, which represents a key finding of this study. In the revised manuscript, these findings of this study and their implications have been discussed in relation to the treatment of SA patients and suicide prevention.

We have added the following statements to the conclusion section to support these points.

In this study, the lack of significant findings supporting the identification theory—such as the absence of correspondence between the sex and age of reported suicide decedents and those of SA patients, as well as the non-significant impact of famous person suicides—suggests that, in the context of routine suicide reports and the widespread adoption of media recommendations, susceptibility is more strongly influenced by individual psychological and regional sociocultural factors of news recipients, rather than by the characteristics of the reported suicide decedents.(Lines 636-642)

Moreover, they (the findings) highlight the importance of tailoring psychoeducational interventions to the unique response profiles of different populations. Building on these findings, future research should explore the factors underlying sex- and age-related differences in media responsiveness across diverse sociocultural contexts, with the goal of optimizing suicide prevention programs and treatment strategies for SA patients. (Lines 646-650)

Comment 2-4

In some parts of the manuscript, particularly in Methods section, there is heavy reliance on jargon and complex terminology that could be simplified for broader audiences, including policymakers and mental health practitioners.

Response 2-4

To ensure a smoother description of the suicide situation in Japan while avoiding the use of "jargon and complex terminology," we revised the terms and added explanations where necessary. First, we replaced the term "murder-suicide" with "homicide-suicide," which has become a more commonly studied research topic in recent years. Although the term "multiple suicide" is somewhat ambiguous and may have varied interpretations, we continued to use it, defining it as incidents involving multiple suicide decedents. Furthermore, we have added an explanation for the term "Shinju," describing it as a homicide-suicide or multiple suicide occurring within particularly intimate relationships. We also included a cultural note explaining that, in Japan, "Shinju" has historically been a theme in theater and literature, contributing to a cultural tendency to empathize with such acts.

…“Jisatsu (suicide)” and “Shinju (homicide-suicide or multiple suicide that occurred within particularly intimate relationships).” "Shinju" is a term unique to Japan and has long been a theme in Japanese theater, literature, and other cultural expressions.(32) (Line 120-122)

Incident characteristics examined included homicide-suicide (cases involving the murder of another person prior to the suicide) and multiple-suicide (cases involving more than one decedent, including serial or group suicides) as prominent features of suicide. (Lines 128-130)

Further changes regarding 'Shinju' will be described in Response 3-15.

Reviewer #3:

Comment 3-1

The Authors should clarify in Method section why specific variables (age, sex) were prioritized over others, as their relevance to broader outcomes is not immediately evident.

Response 3-1

As indicated in the Introduction section, sex and age have been central to previous research. Specifically, the identification theory highlights the correspondence between the sex and age groups of suicide decedents and media recipients as a key issue. To emphasize the importance of these variables, we have added the highlighted phrase below to the list of investigation items in Methods section.

Furthermore, we have organized the order of the investigated items to maintain consistency, and the explanations of the items have been presented in that order.

To investigate article characteristics, we chose items of the sex and age of suicide decedents, which are central to the focus of this study, their social status, occupation, and the suicide methods used. Incident characteristics examined included homicide-suicide (cases involving the murder of another person prior to the suicide) and multiple-suicide (cases involving more than one decedent, including serial or group suicides) as prominent features of suicide. (Lines 126-130)

Additionally, as indicated in Response 2-3, we have enhanced the descriptions in the Conclusion subsection to link the findings of this study more explicitly to the main focus on sex and age group differences, ensuring consistency throughout the manuscript.

Comment 3-2

Certain aspects of Method section lack clarity. For example, the reference to “search terms” raises questions about whether the search was conducted using physical or digital copies of newspapers.

Response 3-2

The search was conducted using digital copies of the newspapers. The term "Jisatsu (suicide)" and “Shinju” were searched across the full text of articles in a news database. This has been explicitly clarified in the Methods section as follows.

A digital search was conducted across the full text of articles using the search terms “Jisatsu (suicide)” and “Shinju (homicide-suicide or multiple suicide that occurred within particularly intimate relationships). (Lines 119-121)

The 'lack of clarity' is presumed to stem from the use of 'Shinju' as a search term, as well as the inconsistent order in presenting and explaining the investigation items. Please refer to Response 2-4 for our actions addressing these issues.

Comment 3-3

The operational definitions of suicide attempts and their severity require further elaboration. Were standardized assessment tools used to classify suicidality beyond the items mentioned in lines 167-168 (beyond the description for items 246 and 250 of SADS)

Response 3-3

Our Consultation Liaison team, who provided clinical DATA of SA patients for the study, did not use standardized assessment tools. Instead, we have added definition of SA eligible for treatment by our hospital’s Consultation Liaison team.

SA patients were individuals hospitalized for the treatment of physical injuries resulting from suicide attempts, which are defined as concrete actions based on suicidal ideation,(33) …(Line 165)

Comment 3-4

The study refers to a Bonferroni correction (in Methods section), but it is unclear whether this adjustment was consistently applied across all analyses. Were adjustments made for multiple comparisons to control for Type I errors?

Response 3-4

Since each suicide incident was reported by two newspapers on average, we applied the Bonferroni correction, making the significance level twice as stringent, based on the rationale that approximately two tests were conducted per incident, and that the analysis aimed to assess the effect of a single article per incident. In contrast, for tests involving sex differences in the data of suicide decedents or suicide incidents, in which duplicate cases were excluded, and therefore, this correction was deemed unnecessary.

The explanation in Response 3-7 would contribute to understanding the use of the Bonferroni correction in handling the article data.

For the analyses of news articles, since each incident was covered slightly less than twice on average, the significance level was set at 0.025 (0.05/2) (two-tailed) using Bonferroni's correction. For the analyses of data with duplication-eliminated incident cases, a significance level of 0.05 was applied. (Lines 212-215)

Comment 3-5

Multiple/murder-suicides represent distinct phenomena. The Authors should provide a stronger rationale for their inclusion in the study to make this situation clear.

Response 3-5

Homicide-suicide and multiple suicide were selected as prominent features of suicide incidents. We have added the following description to clarify this point.

Incident characteristics examined included homicide-suicide (cases involving the murder of another person prior to the suicide) and multiple-suicide (cases involving more than one decedent, including serial or group suicides) as prominent features of suicide. (Line 130)

Comment 3-6

The Authors should provide a rationale for excluding follow-up news reports. Moreover, in cases where the same event was reported by multiple sources, only the first report was considered. However, repeated exposure to an event through multiple sources could be an important factor. Did the Authors account for the potential influence of cumulative reporting on admissions?

Response 3-6

This study focused on the short-term impact within one week after an incident. It was limited to initial reports issued within one week following suicide incidents, and the subjects of the study were SA patients who presented within one week after the reports were released. Follow-up articles were excluded, as they are often published some time after the incident and do not reflect the immediate impact of the incident.

The following descriptions have been added in the manuscript.

This study examined the association between the characteristics of initial suicide reports in four major newspapers and the differences in admission numbers (Δs) of SA patients across sex and age groups during one-week pre- and post-article release periods. (Line 30)

The aim of this study was to explore the relationship between newspaper initial reports of suicide incidents and hospital admissions of SA patients immediately following the release of the report. (Lines 95-96)

To examine the association between the characteristics of initial suicide reports immediately after incidents and the short-term change in SA patient admissions, we selected articles reporting actual suicides within a week of publication, excluding follow-up reports. (Lines 123-125)

Comment 3-7

The terms "article-based data," "incident-based data," and "suicide-decedent-based data" require clearer definitions. Additionally, the structure of the first part of the results, which describes and analyses characteristics of suicides and suicide attempts, should be reorganized to align with these distinctions.

Response 3-7

Incident-based data' and 'suicide-decedent-based data' were used in the preliminary analysis of this study, with the primary analysis focusing on 'article-based data.' 'Incident-based data' was created to present the characteristics of reported incidents while eliminating duplicated incident reports. 'Suicide-decedent-based data' consists of information on all suicide decedents from the duplication-eliminated incident data. All of these data were utilized in the analysis and are also included in the supporting files.

In Discussion section's 'Characteristics of Newspaper Suicide Reports and SA Patient Admissions' subsection, these data were used to elucidate the features of the reports examined in this study by comparing them with other suicide and SA statistics.

In the revised manuscript, we have opted not to use the terms 'incident-based data,' 'suicide-decedent-based data' and 'article-based data.’ Instead, we have provided explanations in Methods section and in the Notes for Tables 1 and 2 as follows.

To avoid duplication when presenting the characteristics of suicide decedents and incidents reported in multiple news articles, data from the earliest article were chosen. Furthermore, if multiple reports covering the same incident were published on the same day, data from the newspaper with the largest circulation were used. (Lines 143-146)

[Restatement] For the analyses of news articles, since each incident was covered slightly less than twice on average, the significance level was set at 0.025 (0.05/2) (two-tailed) using Bonferroni's correction. For the analyses of data with duplication-eliminated incident cases, a significance level of 0.05 was applied. (Lines 203-206)

When multiple newspapers reported on the same incident, to avoid duplication, the suicide decedents presented in this table were those described in either the earliest released report or, in cases of simultaneous reporting, the one from the newspaper with the largest circulation. (Note of Table 1)

When multiple newspapers reported on the same incident, to avoid duplication, the suicide incidents presented in this table were those described in either the earliest released report or, in cases of simultaneous reporting, the one from the newspaper with the largest circulation. (Note of Table 2)

Comment 3-8

A more detailed justification of the statistical methods is needed. The rationale and objectives behind these analyses should be clearly articulated.

Response 3-8

As mentioned in Response 2-2, nonparametric MANOVA was employed to evaluate the association between the characteristics of newspaper reports and each sex and age group’s Δ, while considering the associations with other groups' Δ. Additionally, this method was adopted to enable the analysis of sex and age groups’ Δs as dependent variables, despite their singular matrix.

Comment 3-9

Additionally, effect sizes should be reported where applicable.

Response 3-9

We appreciate the valuable suggestion. The calculation of r, the effect size for the Wilcoxon rank-sum test, was successfully performed using the wilcoxonR function in the rcompanion package. The r values for items with significant findings have been included in the note for Table 7. However, the calculation of effect sizes for the nonparametric MANOVA remains unaddressed due to the lack of established standard methods and the absence of such a function in the MANOVA.RM package.

The following statements have been added to the revised manuscript.

Effect size r for Wilcoxon rank-sum test was calculated using wilcoxonR function in the Package rcompanion (version 2.5.0). (Lines 209-211)

Some suicide methods showed significant differences in Δs for patient groups compared to the reference, even though their effect sizes were less than moderate. (Lines 423-425)

The effect size r for the suicide methods with a significant difference (p < 0.025) compared to the reference were as follows: Firearm discharge for ΔYounger, -0.140; Gas poisoning for ΔFemale and ΔMale, 0.152 and -0.238, respectively; and Other methods for ΔFemale and ΔYounger, 0.240 and 0.249, respectively. (Note of Table 7)

Comment 3-10

The rationale and purpose of the chi-square analyses should be clarified. The placement of another chi-square analysis immediately after the table may be confusing to readers and should be reconsidered without an explanation.

Response 3-10

We considered the differences in sex and age groups regarding the characteristics of news suicide reports and SA patients to be an important reference point for discussing the results of this study. Accordingly, this has been stated in Methods section.

The preliminary stage of the analysis involved using the χ² test to examine sex and age group differences in the characteristics of newspaper articles and admitted SA patients, as this was crucial for interpreting the differences observed in the study results. (Lines 190-192)

We acknowledge that placing the results of the Chi-square analyses of suicide types by sex and age groups immediately after Table 1 may cause confusion for readers. Therefore, in the revised manuscript, we have added Table 2, which presents the basic statistics of suicide types, aligning with the legitimate order of description in the Methods section.

Comment 3-11

Furthermore, some sample sizes in these analyses are quite small, which should be acknowledged as a limitation.

Response 3-11

The comment is absolutely correct. We have added the following statement to the Limitation section: " The fine categorization of variables reduces the number of cases per group, which may affect the study's results by lowering statistical power and increasing the likelihood of type 2 errors." (Lines 612-614)

Comment 3-12

In general, overly complex tables may be difficult to interpret without extensive cross-referencing.

Response 3-12

We agree with the comment. To simplify the tables, we omitted the test statistics for sex and age group differences in Tables 1 and 2.

Comment 3-13

Lines 432 and 525 in Results section (more precisely, ”in Discussion section”), the terms "effect" and "impact" imply causation, which cannot be inferred from the current study. More cautious language should be used.

Response 3-13

The sentence in Line 432 in the previous manuscript are corrected as follows

The finding in the preliminary analyses that reporting on younger suicide decedents was correlated with a decrease in subsequent overall admissions of SA patients suggests that such articles may have a suppressive effect by evoking negative feelings towards suicide or SA. (Line 485)

The sentence in Line 525 of the previous manuscript has been removed in response to Comment 2-3.

Comment 3-14

The study reports a suppression effect for firearm-related suicides, which is surprising especially given the inclusion of murder-suicides. The explanation attributing this finding to strict gun control is not entirely convincing, as fire arms have been associated with violent behaviors even in countries with stringent regulations (e.g., Colasanti et al., 2024, on homicide-suicide in Italy). Further discussion of this finding is warranted.

Response 3-14

It is reasonable to question this finding. However, it can be understood in light of Japan's extremely special circumstances. Japan’s estimated firearm ownership rate is only 0.3 guns per 100 residents, which is exceptionally low compared to other countries. For reference, the global average firearm ownership rate is 6.6 guns per 100 people, while Italy’s firearm ownership rate is estimated to be 12.7 guns per 100 people.

To facilitate readers' understanding of this situation, we have added the following statement.

This discrepancy may be attributed to Japan's long-standing strict gun control policies, which have resulted in an extremely low civilian firearm holding rate of 0.3 per 100 people in 2017, compared to the global average of 6.6. (40) Firearm suicides may be especially feared and stigmatized, potentially contributing to a suppression of SA incidents following the news release on firearm suicide. (Lines 491-495)

Comment 3-15

The non-significant findings regarding the sex and age characteristics of reported suicide decedents are unexpected given prior research. The Authors should explore potential explanations for these results in greater depth.

Response 3-15

We agree with the comment on the necessity of considering the unexpected non-significant findings in greater depth. One such finding is the lack of an observed increase in SA cases following 'famous person suicide,' which has already been discussed in our previous manuscript. Another is the absence of a decrease in SA occurrences after reports of homicide-suicide and multiple suicide, which are strongly associated with negative connotations. One possible explanation we propose is that the cultural sympathy many Japanese feel toward 'Shinju' may reduce the aversion to homicide-suicide and multiple suicide, thereby affecting the possibility of a post-report decrease in admission numbers.

Additionally, the overlap between these two types of suicide incident and the suicide referred to as Shinju, the latter of which is regarded with relatively lenient attitudes in Japan, may partly explain the lack of changes in SA occurrences following reports of these suicides in this study. In Western countries, it has been reported that the majority of individuals committing homicide-suicide are male.(46, 47) However, in Japan, Shinju often occurs within mother-child relationships.(32) The finding in this study that there was no significant difference in sex among the perpetrators of homicide-suicide suggests that many of these cases involved mother-child Shinju. Likewise, the observation that the majority of the reported multiple suicide cases involved female decedents also indicates that many of these incidents may have occurred within intra-family relationships. (Lines 538-548)

Comment 3-16

The weak but significant correlations between Δs and article characteristics (e.g., suicide method) are informative but require deeper interpretation. The Authors should discuss why certain methods show stronger correlations and how these findings align with existing literature.

Response 3-16

Previous studies that systematically examine the association between the suicide methods reported by suicide news articles and the occurrence of suicides are very limited, with only a few examples such as the studies by Synor et al. (2018) and Niederkrotenthaler et al. (2009) Moreover, to the best of our knowledge, no studies have investigated the differential association of the suicide methods reported with the number of SA occurrence across sex and age groups, as done in this study. The findings of the former type of previous research correspond only to the association between ΔWhole and reported suicide methods presented in Table 4 of this study. Therefore, the findings from the previous research are presented in the subsection titled 'Associations Between Newspaper Suicide Reports and SA Patient Admissions' within the Discussion section and are further elaborated upon in the subsequent subsections.

In the revised manuscript, as described in the Responses 3-14 and 3-15, we have added further descriptions of concerning the findings in relation to the previous literature.

Comment 3-17

Studies that rely on newspaper reports have inherent limitations, including biases in reporting—such as the overrepresentation of dramatic or violent suicide methods. While the Authors briefly acknowledge this, a more detailed discussion of these biases is needed. Similarly, the potential impact of the study’s regional focus should be addressed.

Response 3-17

The reporting of newsworthy incidents introduces bias when using media reports as materials for epidemiological research. However, the purpose of this study is to investigate the impact of reports on such newsworthy incidents, and we believe this bias should be addressed in Discussion section rather than in Limitations. In this revised manuscript, the differences between actual occurrences of suicide and SA and the reported suicides are examined in Discussion section's 'Characteristics of Newspaper Suicide Reports and SA Patient Admissions.'

In the revised manuscript, the focus on regional aspects is highlighted as one of the key conclusions of this study and has been referred to in the Subsection 'Implications of Associations between Suicide News Reports and Subsequent SA Patient Admissions' in Discussion section (Line 595), as well as in Conclusions (Line 641).

Comment 3-18

The authors might consider including a figure to visually depict trends in suicide attempt admissions over time, potentially disaggregated by patient group.

Although it may be beyond the scope of this study, future research could explore seasonal variations and conduct time-series analyses.

Response 3-18

This study examines the four variables based on sex and age groups; therefore, we were unable to find an appropriate method to visually depict the results as suggested. We kindly ask for your understanding regarding this situation.

Additionally, the revised manuscript includes the following changes:

1. Added a column for the total of each item in Tables 1, 2, and 3.

2. Created a subsection titled 'Characteristics of Newspaper Suicide Reports and SA Patient Admissions' at the beginning of Discussion section.

3. Added the following sentences in Conclusion subsection.

Suicide method reporting of gas poisoning, firearm discharge, and infrequent methods was associated with change in admission numbers for certain SA patient groups. These associations are likely to be interpretable within psychological and socio-cultural contexts. (Lines 631-634)
