# Supplementary material for: Sex and age differences in the association between routine suicide newspaper reporting and change in admissions of suicidal patients: An investigation at an emergency and critical care center in Tokyo
Source: PLoS One. 2025 Aug 18;20(8):e0330343. doi: 10.1371/journal.pone.0330343 (PMC12360520; doi:10.1371/journal.pone.0330343)
Supplement: Attachment — Submitted filename: Response_to_Reviewers_auresp_2.docx [file pone.0330343.s007.docx]

**RE: PONE-D-24-46119** Sex and age differences in the association between routine suicide newspaper reporting and change in admissions of suicidal patients: An investigation at an emergency and critical care center in Tokyo

**Our responses to comments by reviewers:**

**Reviewer #3:**

**Comment 3-1**

The operational definition of suicide attempts relies on non-standardized, clinician-rated criteria without the use of validated instruments. While the Authors clarify that SADS items were referenced and provide a working definition of SA, the lack of structured assessments undermines the comparability and psychometric rigor of the patient data. This limitation should be explicitly acknowledged in the Methods and Limitations sections, with a note on the potential impact on validity and inter-rater reliability. Furthermore, the provided definition of SA might exclude low-lethality but high-intent attempts, potentially introducing sampling bias.

**Response 3-1**

As the study did not employ standardized assessments for suicide attempts, there is a possibility—just as the reviewer points out—that patients with more severe physical damage due to their suicide attempts were more likely to be included, introducing a potential sampling bias. It is essential to clarify that the findings of this study are based on a patient population selected under such methodological limitations. Therefore, we have made the following corrections.

**Response 3-1-1**

The following correction has been made

**Previous manuscript**

Data from an Emergency and Critical Care Center from 2012 to 2019 were used. (Lines 32-33 in Abstract)

**Revised manuscript**

Data from an Emergency and Critical Care Center from 2012 to 2019 were obtained through a review of medical records. (Lines 32-33 in Abstract)

**Response 3-1-2**

The following correction has been made

**Previous manuscript**

Data on SA patients admitted to the ECC Center from April 1, 2012, to January 31, 2019, were obtained by reviewing medical records (Lines 171-172)

**Revised manuscript**

Data on SA patients admitted to the ECC Center from April 1, 2012, to January 31, 2019, were obtained through a retrospective review of medical records. (Lines 170-171)

**Response 3-1-3**

The following sentence has been inserted in Limitations section.

Fourth, there are limitations associated with the use of medical record review, including the inability to eliminate selection bias in identifying SA patients, to ensure the validity and reliability of assessments, and the lack of direct access to patients’ experiences of receiving media messages. Further research should employ standardized assessments for suicide attempts and conduct interview-based surveys, as in previous studies (38, 39, 48). (Lines 616-621)

**Comment 3-2**

It appears that the Bonferroni correction was applied specifically to analyses based on article-level data, where duplication across reports was retained. At the same time, other sections indicate that duplicates were removed to avoid inflation of incident counts. If this distinction underlies the Authors’ approach, it should be made more explicit. As it stands, the rationale for correction is not clearly aligned with the data handling procedures described elsewhere. I encourage the Authors to review this carefully for consistency and clarify accordingly. In other words, the manuscript toggles between article-level data (where each article is a unit) and incident-level data (where duplicates are removed). The concern is that this distinction is not clearly mapped onto the analysis flow, leaving the reader uncertain about which dataset structure was used for which test, and whether correction thresholds were consistently applied.

**Response 3-2**

While article-level data served as the primary focus of analysis in this study, we also analyzed datasets in which duplications of suicide decedents and reported incidents across articles were removed. This was necessary to describe the characteristics of suicide decedents and incidents, and to allow comparisons with respective statistics of other kind. Because creating these duplication-removed datasets involved somewhat complex procedures, we explained the process in the previous manuscript following the description of newspaper variables and in the notes of Table 1 and Table 2.

For analyses using these duplication-removed datasets, no repeated statistical testing was conducted. However, in the analyses investigating the association between article-level data and SA patient admissions, we applied Bonferroni correction under the rationale that each incident was reported in approximately two articles, effectively leading to repeated tests per incident.

To ensure consistency and clarity in how these datasets were handled, we have made the following revisions to the manuscript.

**Response 3-2-1**

Concerning the use of the Bonferroni correction, additional wording has been added to clarify the rationale.

For the analyses of news articles, since each incident was covered in slightly fewer than two articles on average—effectively resulting in repeated testing—the significance level was set at 0.025 (0.05/2) (two-tailed) using Bonferroni’s correction. (Lines 209-211)

**Response 3-2-2**

We have expanded the explanation of the purpose behind creating the dataset with duplicate reports removed.

**Previous manuscript**

To avoid duplication when presenting the characteristics of suicide decedents and incidents reported in multiple news articles, data from the earliest article were chosen. Furthermore, if multiple reports covering the same incident were published on the same day, data from the newspaper with the largest circulation were used. (Lines 143-146)

**Revised manuscript**

To present the characteristics of reported suicide incidents and suicide decedents separately, two distinct datasets were created, with duplication from multiple newspaper reports on the same incident removed. Specifically, data from the earliest published article were selected; furthermore, if multiple reports on the same incident were published on the same day, the report from the newspaper with the largest circulation was used. (Lines 141-145)

**Response 3-2-3**

We adopted a consistent principle throughout the manuscript to present the dataset description, analyses, and results in the order of suicide decedents, incidents, and articles. The following is the revised explanation of the processes for constructing the incident and article datasets, revised in accordance with this principle.

Incident types primarily consisted of Famous person suicide, Homicide-suicide, and Multiple suicide. Incidents that did not fall under any of these types were categorized as unclassified type. When incident types were treated as a single categorical variable, those that fell under more than one type were classified as combination type.

Regarding suicide news articles, minor adjustments were made to prepare the data for analysis. To accurately rate incidents involving more than one suicide decedent, 2-value (0, 1) variables related to sex and age—Female decedent included and Younger decedent included (defined as a decedent equal to or younger than the median age)—were created. When suicide decedents used different suicide methods within an incident, the less frequent one was used operationally for classification. (Lines 146-154)

We also revised the order of presentation to follow the sequence of suicide decedents, incidents, and articles, as shown in the example below.

“reported incidents and suicide decedents” **→** “reported suicide decedents and incidents” (Line 190)

**Response 3-2-4**

The significance threshold (p < 0.025) was added to the descriptions of the results for the comparisons between admission numbers during the one-week periods before and after article release for each patient group, as well as for the correlation analyses between the Δs and article characteristics, to clarify that these sections pertain to article-level analyses to which the Bonferroni correction was applied.

The significance of the increase in admission numbers across all patient groups far exceeded the designated level (p < 0.025). …(Lines 336-337)

Weak but significant correlations (p < 0.025) between Δs for patient groups and article characteristics were as follows … (Lines 343-344)

**Comment 3-3**

SA patients were divided into "Younger" and "Older" groups based on whether they were below or above the median age of suicide decedents. While this choice is theoretically justifiable, especially if guided by identification theory, which emphasizes alignment between media representations (decedents) and recipients (patients), the rationale is not clearly explained in the current manuscript. I recommend that the Authors add a sentence explicitly clarifying that this cutoff was chosen not to reflect the patient population distribution, but rather to test age-related susceptibility to suicide reporting effects. In other words, the Authors should clarify this as a theory-driven choice.

**Response 3-3**

The distinction between younger and older groups of SA patients and reported suicide decedents was made to examine the association between patient age groups and the basic characteristics of suicide news articles, with particular attention to the age groups of the suicide decedents. Ideally, the analysis would be stratified into more detailed age groups; however, due to limited statistical power, we opted for this broad categorization as a pragmatic approach.

**Response 3-3-1**

The limitation that the age groupings were too broad to allow clear conclusions has been revised in the Limitations section as follows.

**Previous manuscript**

Fourth, the fine categorization of variables reduces the number of cases per group, which may affect the study's results by lowering statistical power and increasing the likelihood of type 2 errors. (Lines 612-614)

**Revised manuscript**

Fifth, some analyses may have been affected by limited statistical power. For example, fine categorization reduced group sizes, increasing the risk of type II errors, and broad age groupings of reported suicide decedents and SA patients constrained the clarity of conclusions. (Lines 621-624)

**Response 3-3-2**

Because the results regarding age grouping cannot be interpreted definitively, we replaced "absence" with "lack" in the sections below.

The lack of evidence supporting the identification theory in this study may be due to its focus on routine (low impact) suicide reports …(Lines 589-590)

In this study, the lack of significant findings supporting the identification theory—such as evidence of correspondence between the sex and age of reported suicide decedents and those of SA patients,… (Lines 645-647)

**Comment 3-4**

The Authors state that follow-up news reports were excluded in order to focus on the short-term impact of initial suicide coverage within a one-week period. While this decision aligns with their analytic design, it does not fully address the concern about cumulative exposure. A growing body of literature suggest that repeated exposure to suicide-related content (over time or by multiple sources) can compound risk, particularly among vulnerable individuals. By excluding follow-up reports and analysing only the first article per incident, the Authors may inadvertently underrepresent the real-world exposure profile of individuals who consume multiple media reports on the same suicide event. While I appreciate the clarity of the short-term focus, the ecological validity of the findings is limited. The Authors should consider explicitly acknowledging this limitation or potential future direction in the manuscript, as it would strengthen the transparency of the study’s conclusions.

**Response 3-4**

Our decision to exclude follow-up suicide reports was primarily intended to simplify the evaluation of the articles analyzed, while capturing the most impactful coverage by focusing on initial reports. However, this selection strategy inevitably excludes other types of reporting, such as follow-up coverage, and should be acknowledged as a limitation.

**Response 3-4-1**
The limitation arising from restricting the target suicide reports to initial newspaper articles has been added to the Limitations section as follows, and the numbering of the subsequent limitations has been adjusted accordingly:

Second, selecting only initial news articles for analysis to achieve a simplified data structure introduces some problems, including the inability to assess the cumulative effects of follow-up reports. Future research should aim to incorporate and analyze other types of coverage. (Lines 611-614)

**Additional correction 1**

We have revised the short title as follows.

“Suicide Reporting; Effects on Sex and Age of ER Patients” **→** “Effects of Suicide News Reporting on Sex and Age of ER Patients.” (Line 5 in Abstract)

**Additional correction 2**

In the revised Abstract, the discussion was focused on the findings from the MANOVA. The following is the opening sentence of the Discussion subsection."

**This study demonstrates a differential impact of suicide news articles reporting gas poisoning, infrequently reported suicide methods, and firearm discharge, on SA patient admissions across sex and age groups. (Lines 47-49)**

**Additional correction 3**

In the previous manuscript, the study period for the suicide report collection was set as “(the period) between April 1, 2012, and January 31, 2019.” However, since the number of SA patient admissions during the one-week periods before and after article publication needed to be assessed, the correct study period should have been “(the period) between April 7, 2012, and January 25, 2019.” This point has now been corrected:

“(the period) between April 1, 2012, and January 31, 2019” (Line 117) → “between April 7, 2012, and January 25, 2019” (Lines 116-117)

**Additional correction 4**

The following sections have been revised using semicolons to ensure consistency with other parts of the manuscript.

Significant differences by sex and age group were found, as follows: younger decedents (≤ 35 years) included a higher percentage of females; females were less represented among occupational or social status groups of Famous persons, Public workers, Police officers or SDF members, and Criminal or suspected persons, as well as in cases involving Hanging or Firearm discharge; in contrast, females were more represented in cases involving Height jumping and Gas poisoning.

Younger decedents accounted for a smaller percentage of Famous persons, Public workers, and Professional experts; in contrast, they accounted for a higher percentage in cases involving Railroad jumping and Height jumping, and a lower percentage in cases involving Hanging, Firearm discharge, and Cutting or stabbing. (Lines 261-269)

Significant associations between suicide incident types and sex- and age-related characteristics were observed, as follows: Female decedent included was negatively associated with Famous person suicide and positively associated with Multiple suicide; Younger decedent included was negatively associated with both Famous person suicide and Homicide-suicide. (Lines 282-285)

**Additional correction 5**

The first paragraph of the subsection "Associations between Reported Suicide Methods and Sex and Age Group Admissions" in the previous manuscript introduced studies that addressed the overall impact of reported suicide methods on news recipients, rather than the differential impact across sex and age groups. Therefore, it has been moved to the earlier subsection "Associations Between Newspaper Suicide Reports and SA Patient Admissions," and a discussion has been added regarding the findings presented there.

It was recognized that reporting on other suicide methods was not associated with changes in the overall number of admissions. By contrast, in previous reviews on suicide cases, (1, 5, 7, 11, 13, 15, 27) have reported that media coverage of specific suicide methods influenced the occurrence of suicides. Some studies have found that certain methods, such as jumping from a height, (8, 14, 23) are associated with an increase in suicide incidents, whereas others, such as cutting or stabbing, and railroad jumping,(23) may be associated with a decrease in suicide rates.

This discrepancy may be explained by differences in the research focus—specifically, whether the outcome examined was suicide or suicide attempt—and by variations in the socio-cultural backgrounds of the study settings. (Lines 500-508)

In conjunction with this change, the explanation preceding this paragraph regarding the exceptional nature of firearm suicides in Japan was replaced with the following sentence.

The primary reason for this discrepancy may lie in Japan's unique circumstances of long-standing, strict gun control policies, which have led to an exceptionally low civilian firearm possession rate of 0.3 per 100 people in 2017, compared to the global average of 6.6. (Lines 489-492)

**Additional correction 6**

The following correction and addition **were** also made to the note of Table 1.

^d^ Of the suicides by gas poisoning, 64 cases (88.9%) involved charcoal burning (CO poisoning). (Line 253)

^e^ Among the suicides by firearm discharge, 15 cases (22.1%) were committed by police officers or members of the Self-Defense Forces (SDF). (Lines 254-255)
